# Impact of Physical Activity on Cognitive Decline, Dementia, and Its Subtypes: Meta-Analysis of Prospective Studies

**DOI:** 10.1155/2017/9016924

**Published:** 2017-02-07

**Authors:** Chris B. Guure, Noor A. Ibrahim, Mohd B. Adam, Salmiah Md Said

**Affiliations:** ^1^Department of Biostatistics, School of Public Health, University of Ghana, Legon, Accra, Ghana; ^2^Department of Mathematics, Faculty of Science, Universiti Putra Malaysia, Serdang, Selangor, Malaysia; ^3^Institute for Mathematical Research, Universiti Putra Malaysia, Serdang, Selangor, Malaysia; ^4^Department of Community Health, Faculty of Medicine & Health Sciences, Universiti Putra Malaysia, Serdang, Selangor, Malaysia

## Abstract

The association of physical activity with dementia and its subtypes has remained controversial in the literature and has continued to be a subject of debate among researchers. A systematic review and meta-analysis of longitudinal studies on the relationship between physical activity and the risk of cognitive decline, all-cause dementia, Alzheimer's disease, and vascular dementia among nondemented subjects are considered. A comprehensive literature search in all available databases was conducted up until April 2016. Well-defined inclusion and exclusion criteria were developed with focus on prospective studies ≥ 12 months. The overall sample from all studies is 117410 with the highest follow-up of 28 years. The analyses are performed with both Bayesian parametric and nonparametric models. Our analysis reveals a protective effect for high physical activity on all-cause dementia, odds ratio of 0.79, 95% CI (0.69, 0.88), a higher and better protective effect for Alzheimer's disease, odds ratio of 0.62, 95% CI (0.49, 0.75), cognitive decline odds ratio of 0.67, 95% CI (0.55, 0.78), and a nonprotective effect for vascular dementia of 0.92, 95% CI (0.62, 1.30). Our findings suggest that physical activity is more protective against Alzheimer's disease than it is for all-cause dementia, vascular dementia, and cognitive decline.

## 1. Introduction

At the end of 2015, it was estimated that about 46.8 million people across the globe lived with Alzheimer's disease and other dementia subtypes [1]. More importantly, 74.7 million people will be demented by 2030 and 131.5 million by end of 2050 with an increase of 68%. The United States of American (USA) alone is projected to record about 7.1 million people with dementia by 2025 and 13.8 million by end of 2050 [2]. Individuals above the ages of 65 accounted for 5.2 million while those lower than that accounted for 200,000 dementia cases at the end of 2015. The estimated total cost for people living with dementia and its subtypes worldwide according to Prince et al. [1] stands at $818 billion and is projected to rise to about $1 trillion in the next three years and then to $2 trillion by 2030. The survival rate of individuals diagnosed with dementia and its subtypes after the age of 65 where it is more prevalent stands at just 4 to 8 years on the average [3, 4]. The sixth most deadly disease in the USA in recent times is Alzheimer's disease for all ages but the fifth for people whose ages are greater than 65. It is also the leading cause of morbidity and disability with 61% likely to die before the age of 80 as compared to 30% for the nondemented [5].

Cognitive decline is observed to be one of the warning signs of the onset of mild cognitive impairment or Alzheimer's disease. Individuals with cognitive decline do experience most of the time memory loss, for instance, forgetfulness and ones inability to function normally or objectively as they used to do [6, 7].

Dementia according to DSM-5 [8] is a serious neurocognitive or degenerative disorder. It interferes with cognitive function and the ability of people to perform day-to-day activities without help. Dementia is observed to be a general term that encompasses different types of distinct symptoms and brain abnormalities [9]. These include, but not limited to, (1) Alzheimer's disease which accounts for about 60–80% of dementia cases in the Western world: Alzheimer's disease involves individual's inability to remember recent events, poor judgement, difficulty walking and speaking, and so on; (2) vascular dementia, which contributes about 10% of all dementia cases and involves ones inability to take decisions, plan, and organise [8]. It was the leading cause of dementia in Japan [10].

There are two types of risk factors associated with cognitive decline, dementia, and its subtypes; these are modifiable and nonmodifiable. The nonmodifiable factors that are more devastating include age, family history, and Apolipoprotein E (APOE)-e4 gene [2, 11]. The modifiable ones according to Moore et al. [12] are seven; these include cardiovascular disease and its risk factors (obesity in midlife, smoking, and diabetes), [13]. Type 2 diabetes has the potential of doubling the incidence of dementia, Alzheimer's disease, and vascular dementia when diagnosed earlier in midlife [14]. Hildreth et al. [15] state that, due to the strong resistance of insulin in obese people, there is a very high increased risk of developing cognitive impairment among people living with obesity. Depression has been identified to be one of the modifiable risk factors and it increases the risk of developing Alzheimer's disease by twofold [16]. Others include education, social and cognitive engagement, and physical activities.

A study conducted by Baumgart et al. [17], with a population-based prospective sample, revealed that there is sufficient evidence to suggest that modifiable risk factors have an effect on dementia and cognitive decline. Baumgart et al. [17] in their findings concluded that physical activity and the ability for individuals to manage cardiovascular risk factors reduces the risk of cognitive decline which may also reduce the risk of dementia. Others whose separate findings support these same conclusions are [18–21]. Prospective studies by other authors that proved contrary to the above findings include [22–25]. According to the latest findings by Kishimoto et al. [23], physical activity reduces the risk of Alzheimer's disease about 49% but played no effective role in all-cause dementia, vascular dementia, and other types of dementia among Japanese elderly.

There are a number of meta-analyses conducted previously in this area but either concentrated on high or moderate against low physical activity on cognitive decline and/or dementia; see, for instance, [26, 27]. A more recent study by Blondell et al. [28] did not differentiate between all-cause dementia and Alzheimer's disease. Though all these meta-analyses have contributed in one-way or another in elaborating on the effects of physical activity to either cognitive decline or dementia, we observed some limitations to their statistical approaches. The limitations are that their analyses were carried out using the frequentist random or fixed effects model and made strong assumptions of normality even with small samples. These studies also made use of the funnel plot by Egger et al. [29] to assess for the presence or otherwise of publication bias which according to Higgins et al. [30] has low power in detecting true effect if it exists especially with small number of studies.

In light of these limitations of the above previous research, we have conducted our work based on the Bayesian methodological framework. Our analysis employed the Bayesian nonparametric approach which does not assume that the study effects follow any particular distribution of interest. This work also used the Copas selection model, Copas and Shi [31], to assess and account for publication bias. To the best of our knowledge, this is the first work to conduct meta-analysis of prospective studies by combining both the Bayesian parametric (Copas selection model) and nonparametric (Dirichlet process) approaches which assess and account and control for publication bias and heterogeneity. It is also the first study to investigate the association between high and moderate physical activities and the risk of cognitive decline, vascular dementia, Alzheimer's disease, and all-cause dementia. Lastly, we have developed a new Bayesian prior distribution for the parameters of the Copas selection model.

The main objective of this study is to ascertain the effects of high and moderate physical activities on all-cause dementia, Alzheimer's disease, vascular dementia, and cognitive decline via systematic reviews and meta-analysis of prospective cohort studies. We believe this will help to settle controversies or conflicting issues surrounding the effects of physical activities on dementia and its subtypes via Bayesian meta-analysis that has a greater power of detecting statistically significant effects if they exist.

## 2. Methods

### 2.1. Inclusion and Exclusion Criteria

Studies that met the inclusion criteria for the outcomes of interest (1) reported either Alzheimer's disease, vascular dementia, or dementia or all, (2) reported cognitive decline or cognitive impairment or no dementia, and (3) had a follow-up of at least twelve months with a baseline screening for the presence or otherwise of Alzheimer's disease, vascular dementia, dementia, and cognition. Studies with less than 12 months' follow-up were excluded mainly because the diseases are rare and 12 months may not be sufficient to actually observe an individual's real change in cognition. Studies less than 12 months may also not have enough samples which has the potential of introducing bias into our analysis. Studies where the primary or secondary exposure outcome variable was physical activity either as a composite score, binary, or categorical at baseline were considered. Cross-sectional and case-control studies were excluded due to the neurodegenerative nature of the outcome variables of interest which has the potential to bias one's ability to recall events. We excluded the clinical presence of a cohort with Parkinson's disease on studies identified at baseline. Also excluded were randomised controlled trials, studies that involved interventions of any kind, and results that were not reported in either odds or hazards ratios. Conference papers were also excluded on the basis that they did not give enough evidence on participants recruitment, appropriate statistical analysis, how the outcome and/or primary exposure variables were measured, and information as to whether outcomes of people who withdrew from the study were included in the analysis or not.

### 2.2. Data Extraction

Studies that met the inclusion criteria as illustrated above were extracted irrespective of country, author, and publication year. Two reviewers separately or individually checked all titles and abstracts of articles or records that were identified through the search process and included all potentially relevant articles. In cases of uncertainty, both reviewers came together and a determination was made as to whether to include or not a particular study. The reviewers met fortnightly for the three months' duration of the work. Throughout the study, the reviewers did not solicit the services of a third party since all issues emanating from the review were amicably solved by them. The reviewers appraised the methodological quality of each of the studies that met the inclusion criterion; refer to Tables  7 and 8 in the supplementary materials for details (in Supplementary Material available online at https://doi.org/10.1155/2017/9016924). We used the Meta-Analyses of Statistics Assessment and Review Instrument (JBI-MAStARI) developed by [32] and presented in Table  8 in the supplementary materials. Using this instrument, studies that scored ≥7 were deemed to be of high quality, those within 4≥ studies ≤6 were judged to be of moderate quality, and that of scores <4 were of low quality. Some but not all the studies reported their results of the exposure variable (physical activity) comparing high to low and moderate to low. Results for high against no/low and moderate against no/low were extracted separately and used as such. Data were obtained from records that reported odds, risk, or hazard ratios as measures of association. The outcome variables are considered as rare and therefore these measures of association are approximately equal. The following were the characteristics of interest upon which data were extracted: authors and year of publication, country of prospective study, year of study, sample size, sex or gender, follow-up years, age of the participants at baseline, methods used to assess Alzheimer's disease, dementia, cognitive decline, and physical activity; for more details, refer to Tables  4 and 5 on the supplementary material. The following instruments were used to examine at baseline and subsequent visits the presence or not of dementia and its subtypes: National Institute of Neurological and Communicative Disorders and Association Internationale pour la Recherche et l'Enseignement en Neurosciences (NINDS-AIREN) criteria and Diagnostic and Statistical Manual of Mental Disorders (DSM) criteria. Questionnaires were used to measure the exposure variable.

### 2.3. Search Strategy

**Figure 1:**
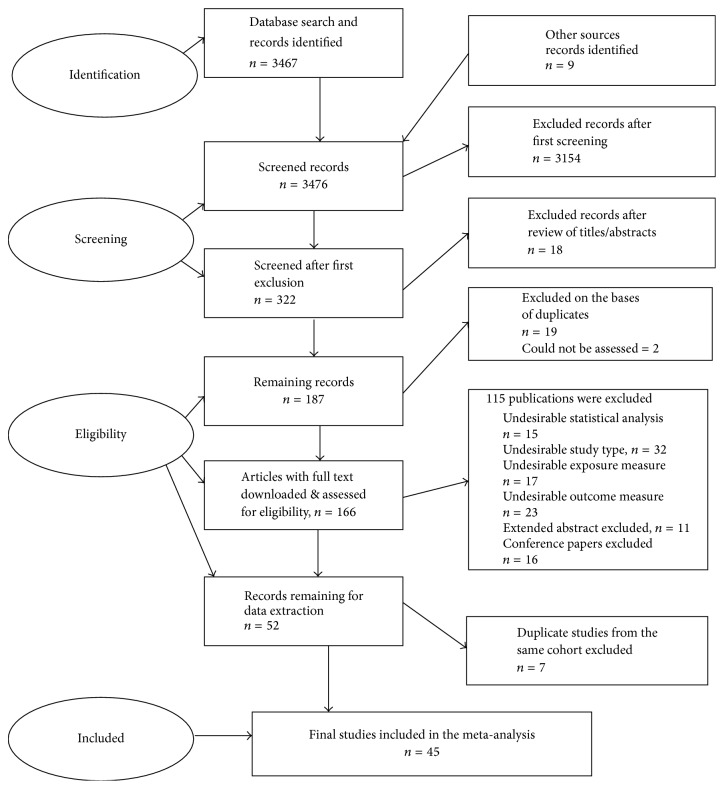
PRISMA flowchart for search strategies.

Electronic databases such as PubMed, Science Direct, Embase, Web of Knowledge, PsychINFO, and Google Scholar were searched for all relevant records necessary for our study. From these databases, the initial search resulted in 3474 studies based on the search criteria. After having identified the studies and screened and assessed their eligibility, the final number that required a complete record download and further screening was 166. Out of the 166 records retrieved, 15 studies implored an undesirable statistical analysis with 49 inapt exposure measures and study types and sixteen conference papers. There were eleven extended abstracts and twenty-three unsuitable study types identified. Other reviews that are not up to date but examined physical activity and either dementia or cognitive decline and were retrieved are [26–28]. The final records that met the inclusion criteria were 45 after removing 7 studies from the same cohort as those already included. Details of the search strategy are illustrated in the PRISMA flowchart, Figure 1 according to Moher et al. [33], and Table  6 provided in the supplementary material contains the checklist. The search retrieved studies conducted across the world, particularly Sweden, Japan, Canada, Finland, Australia, United Kingdom, USA, Iceland, Nigeria, South Korea, Italy, Germany, France, China, Singapore, and Netherlands. There were 45 prospective studies with 58 cohorts records retrieved. Keywords according to Medical Subject Heading terms (MESH) with Boolean operators were implored via the following text words: “physical activity” OR “physical exercise” OR “exercise”, OR “fitness” OR “training” for physical activity. Those of cognitive decline were “cognitive decline” OR “cognitive function” OR “cognitive impairment” OR “cognitive loss” OR “cognition” OR “cognit^*∗*^”, while those of dementia were “dementia” OR “Alzheimer's disease” OR “vascular dementia”. These search strategies retrieved different records which were combined with the Boolean operator “AND" to obtain the first number of records. The search was limited to only prospective cohort and epidemiological studies. There were no restrictions on language and continent or country. Further reviews of the references of all relevant retrieved records were manually conducted and all those that met the inclusion criteria were included.

### 2.4. Group and Subgroup Comparisons

Analyses were carried out using group and subgroup comparisons. First of all, holistic analyses of studies obtained under all-cause dementia, Alzheimer's disease, vascular dementia, and cognitive decline were carried out. These outcomes were subcategorised according to the categorisation of the exposure variable (physical activity). Information on how physical activities were measured on each of the records retrieved are documented and included in the supplementary materials. Briefly, physical activity was measured across the records using self-administered questionnaires where participants were asked how many times they engaged in either daily and/or weekly exercise. Some authors measured physical activity in minutes per day, types of exercise each participant was engaged in, how regular they exercised, and how intense it was. These pieces of information were either dichotomised into no/low and yes/high or categorised into tertile, no/low, moderate and high, or vigorous. For instance, Tolppanen et al. [34] categorised it into three with the question “how often do you participate in leisure time physical activity that lasts at least 20–30 minutes and causes breathlessness and sweating?” Responses according to Tolppanen et al. [34] were “(1) ‘daily'; (2) ‘2-3 times a week'; (3) ‘once a week'; (4) ‘2-3 times a month'; (5) ‘a few times a year'; and (6) ‘never due to illness or injury.' Because of low frequencies in some response categories, midlife leisure time physical activity was recoded into a three-category variable indicating high (responses 1 and 2), moderate (responses 3 and 4), and low (responses 5 and 6) levels of leisure time physical activity in midlife.” Study by Kishimoto et al. [23] defined physically active as people who engage in exercise for at least once or more times per week during leisure time, and these were then divided into two groups: the active group and the inactive group. These were based on information obtained using light or brisk walking, calisthenics, gateball, golf, dancing, jogging, hiking, bowling, cycling, hunting, gardening, and Japanese traditional dance (Nihon Buyo).

Results from this study were obtained for these categorisations and analysed as such in order to observe the effect of high and moderate against no or low physical activities. Mostly, authors report results based on high physical activity alone ignoring moderate PA; see, for instance, [27]. A further subcategorisation was based on sex upon which the results were reported. It was observed that quite a number of the studies such as [35–41] reported separate results for the sex variable. Others include [34, 42–48] and the rest were on both sexes. These sex categorisation analyses were carried out for all the outcomes, that is, Alzheimer's disease, dementia, and cognitive decline except vascular dementia. Further analyses based on duration of individual studies were done. The groupings were on two categories, those with a mean follow-up of ≤5 and >5. The last subcategorisation analysis was on sample size. Studies that had samples greater than 1000 and those with less than 1000 were all subgrouped. There was no specific reason for the justification of this sample size categorisation apart from trying to obtain a balanced number of studies for each group and also just for sensitivity analysis. Also longitudinal studies do involve a lot of samples; hence, it may be reasonable for these categorisations.

#### 2.4.1. Bayesian Parametric Hierarchical Models

It is very common to see meta-analysis from the frequentist methodological approaches applying a graphical approach called funnel plot to visually examine publication bias in systematic reviews and meta-analyses. Sometimes these visualisations are problematic and can easily be misinterpreted to mean that either publication bias is present or not. Though there are other types of hypothesis testing that maybe used to confirm or not its presence, those methods have their short falls especially with small samples. In majority of cases, meta-analysis involves small number of studies of which the use of these methods is not recommended [49]. According to Higgins et al. [30], the funnel plot test generally has low power and therefore a symmetry funnel plot does not necessarily rule out the presence of publication bias. As a rule of thumb, Higgins et al. [30] proposed that the funnel plot should only be used to assess for publication bias when only the number of studies involved is at least 10. In the Bayesian perspective, small samples can easily be handled especially when prior knowledge about the study is present.

Due to the shortcomings of the frequentist methodological approach, this current study applied the Copas selection model to assess and account for publication bias. According to Sutton et al. [50], publication bias exists because most published works are likely to report significant rather than insignificant results. There is always the need to attempt meta-analysis to assess the presence or otherwise of publication bias [50]. The issues of how to deal with publication bias when carrying out meta-analysis have been dealt with by [50]. Apart from publication bias, heterogeneity among studies can also pose a fundamental problem when studies are combined across different geographical locations. A Bayesian nonparametric (Dirichlet process) model is used to control for between-study variation (heterogeneity).

The Copas selection model is viewed as an extension of the usual random effects model that is generally used in meta-analysis when among-study variability is suspected to be present. The model is, therefore, a combination of the random effects and the selection models and can be represented as

(1) yi~Nμi,σi2,μi~Nd,τ2,zi~Nωi,1Izi>0,ωi=α+βsi.

From (1), *μ*_*i*_ takes on a probability model which is seen to be estimating individual study effects deduced to be normally distributed with mean *μ* and variance (between-study variability) *τ*^2^. The assumption for normality is a strong one; as such, we have instead assumed that these study effects *μ*_*i*_ are not necessarily Gaussian distributed; hence, a Bayesian nonparametric approach is implemented alongside the Copas selection model. Refer to the next section for more details. The selection model assumes *ρ* = corr (*y*_*i*_, *z*_*i*_), where *ρ* indicates that the propensity for selection is associated with the observed effect size. Large positive values of *ρ* are related to large positive values of the propensity score *z*_*i*_ given that *y*_*i*_ is large. Therefore, the meta-analysis of the available studies is likely to overestimate the actual effect size provided positive values of the effect size show a better treatment effect which according to Copas and Shi [31] is an indication of publication bias.

These expressions are based on the dependence of the probability of selection on the outcome variable *y*_*i*_ and its standard error *s*_*i*_. The probability that a study will be selected given its standard error is Φ(*ω*_*i*_) = *α* + *β*/*s*_*i*_. The total number of published and unpublished studies according to [51] is ∑_*i*_1/*P*(*z*_*i*_ > 0∣*s*_*i*_). Sampling from the joint distribution can be obtained by first sampling from the propensity score which is a truncated normal distribution with *z*_*i*_ ~ *N*(*ω*_*i*_, 1), where *ω*_*i*_ is the mean of *z*_*i*_ given as *α* + *β*/*s*_*i*_ and variance 1.

Copas and Shi [31] proposed that three out of the five parameters be estimated while the remaining two *α* and *β* are fixed based on the preference of the researcher. In the Bayesian inference, all unknown parameters are random; following Mavridis et al. [51], we assign to these parameters prior distributions. Assume that *ω*_*s*(max)_ ~ Φ^−1^(*P*_low_) and *ω*_*s*(min)_ ~ Φ^−1^(*P*_large_), with *ω*_*s*(max)_ indicating that the smaller the sample size the larger the standard error, hence the lower the probability of the study being published and *ω*_*s*(min)_ vice versa. The probability that a study with smaller or larger standard error will be published can be obtained as *P*_low_ ≤ *P*(*z*_*i*_ > 0) ≤ *P*_large_, where *P*_low_ and *P*_large_ each contains both lower and upper bounds with *P*(*z*_*i*_ > 0) being the probability that a particular study will be selected. It is therefore easy to assign prior distributions to the Copas model parameters *α* and *β* indirectly through *P*_low_ and *P*_large_.

#### 2.4.2. Bayesian Nonparametric Hierarchical Models

The Bayesian random effects model assumes that the study specific effects follow a normal or *t* (in rare cases) distribution. The assumption of normality for study specific effects may not be theoretically justifiable and therefore can be viewed as a very high subjective opinion. A study conducted by Burr et al. [49] has provided enough evidence to suggest the presence of nonnormality for study effects among independent candidate gene studies and also in the presence of high among-study variability similar to this study.

Since the normality assumption without theoretical justification may be viewed as very strong, it is therefore important to relax this assumption and assume that these individual study effects do not follow any particular type of parametric distribution by specifying rather a nonparametric distribution. Consider a situation where the study effects *μ*_*i*_ follow a Dirichlet process which is given as follows:

(2) $$\begin{array}{c} \mu_{i}\;|\;G\sim G,\;i=1,\ldots ,m,\;\;G\sim DP\left(h,G_{0}\right),\;\;G_{0}\sim N\left(0,\tau^{2}\right),\;\;\tau \sim Unif\left(0,2\right),\;\;h\sim Unif\left(0.1,10\right). \end{array}$$

From (2), the assumption is that *μ*_*i*_ comes from the distribution of *G*, where *G* is observed to be a distribution of distributions. *G*_0_ is the baseline also known as the “centre” of the distribution which we assume to follow the Gaussian or normal distribution such that, for any given say, *θ* we have *E*[*G*(*θ*)] = *G*_0_(*θ*), and *h* is the concentration parameter. The concentration parameter determines our a priori belief about *G*_0_. See Sethuraman and Tiwari [52] for details. *τ*^2^ is the usual among-study variability or heterogeneity. We specify a uniform distribution via the standard deviation with parameters 0 and 2. These values are highly subjective and are based on our belief that none of the study specific effects standard deviations can exceed 2. There are different approaches to implementing the DP distribution; see, for instance, [53–56]. In our analysis, we make use of the stick breaking approach as proposed by Sethuraman and Tiwari [52] for its appealing nature. The stick breaking approach by Sethuraman [57] has the following algorithm: generate a set of atoms, *μ*_*i*_^*∗*^ ~ *G*_0_, and that of a set of weights say *w*_*i*_ = *y*_*i*_∏_*j*<*i*_(1 − *y*_*i*_), where *y*_*i*_ are independently and identically distributed with *y*_*i*_ ~ Beta(1, *k*), *i* = 1,…, *∞*. Hence, *G* = ∑_*i*∈*N*_*w*_*i*_*Iμ*_*i*_^*∗*^. The prior for the overall or grand mean effect was *μ* ~ *N*(0,100^2^).

#### 2.4.3. Prior on Copas Model Parameters

The Copas selection model is based on sensitivity analyses as pointed out by Copas and Shi [31]. Since sensitivity analysis yields different posterior summaries, it makes the interpretation of results difficult for researchers due to different estimates for the same parameter. To overcome this limitation of the Copas model, we developed what we refer to as* triangular prior* for the probabilities of publication (*P*_low_ and *P*_large_). These priors specifications do not require (1) the solicitation of information from experts, (2) gathering of information from registry, and (3) guessing values arbitrarily to conduct sensitivity analysis. The prior is obtained by assuming that if *P*_low11_ ~ Unif(0,1) and $P_{low1}=(\sqrt{P_{low11}})$ then the probability of *P*_low_ = (1 − *P*_low1_), and the probability of large is obtained as *P*_large_ ~ Unif(*P*_low_, 1). These formulations follow the logic of a triangular distribution with *f*(*l*, *u*) = 2, 0 ≤ *l* ≤ *u* ≤ 1 and 0 otherwise. The lower and upper bounds are assigned *l* and *u*, respectively. The condition for using the Copas model is that at all times the probability of large must always be greater than the probability of low of which our prior fits. A prior of *ρ* ~ Unif(−1,1) was placed on the correlation coefficient which, according to Copas and Shi [31], is used to determine whether they is any association between the outcome variable *y* and the propensity score *z*, which indicates the presence or otherwise of publication bias.

Two MCMC chains of 45,000 iterations were run with the first 5,000 iterations discarded as burn-in to achieve convergence. This burn-in that was discarded was determined by examining diagnostics such as trace and density plots, Brooks-Gelman-Rubin convergence plots and statistic following Brooks and Gelman [58], and autocorrelation plots. After convergence was achieved, a further 10,000 iterations were performed before posterior summaries were obtained. The* OpenBUGS* software was used for all the analyses in this study. Codes are available and will be provided upon request made to the corresponding author.

## 3. Results

### 3.1. Dementia

The search obtained a total of 25 prospective records with 32 cohorts for all-cause dementia. We calculated an overall estimate of the effect size based on high against low/no physical activity on one side and that of moderate against low/no physical activity on the other. The total number of studies included for the high to low PA was all the 32 cohorts. Out of the 25 prospective studies only 10 clearly defined and examined moderate to low/no physical activity with 15 cohorts. The sample size from the studies ranged from 147 to 4761 with a total of 46909 observations.

**Figure 2:**
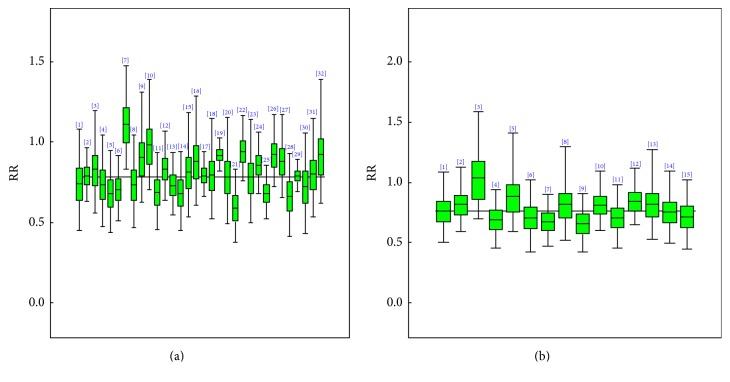
Posterior box plots describing the 95% credible intervals for each study's specific odds ratio (OR) estimate where (a) represents high and (b) represents moderate against no/low physical activity for dementia.

An association of high and moderate against low physical activities was observed for dementia. The overall or grand mean effect and credible interval for high and moderate compared to no/low physical activity are presented in Table 1. From Table 1 and Figure 2, highest physical activity (PA) compared with no/lowest was 0.79 (0.69, 0.88) and moderate PA was 0.76 (0.61, 0.94). This represents a reduction rate of 21% for high PA and 24% for moderate. The presence of publication bias as depicted by the Copas selection model parameter *ρ* was 0.07 (−0.94, 0.96) 95% CI and 0.13 (−0.90, 0.97) for both high and moderate PAs, respectively, an indication of negligible publication bias. The posterior probabilities were 0.55 and 0.59, respectively. We observed a negligible variability between studies for both high and moderate against no/low PA. The estimated *τ* were 0.12, 95% CI (0.03, 0.32) for high and 0.06, 95% CI (0.00, 0.22) for moderate. Sensitivity analysis for highest physical activity against lowest were also conducted and presented in Table 1. Observations from these analyses showed that men had a very little protective effect of 23% over women, who had 22%. A follow-up period of less than or equal to 5 years were 22% protective and those greater than 5 years were 20%. For the sample size, those with more than 1000 had a 21% reduced risk of dementia compared to those with less than 1000 with 22%. There is a significant risk reduction effect (26%) of physical activity for people beyond the age of 65 developing all-cause dementia while insignificant for people below the 65 age group.

### 3.2. Alzheimer's Disease

A total of 17 prospective studies with 21 cohorts examined whether physical activity has any influence on Alzheimer's disease. Most of the studies that examined Alzheimer's disease also examine incidence of all-cause dementia. All the 21 records examined high or vigorous against low or no physical activity but 8 prospective studies with 12 cohorts assessed moderate against low or no physical activity. The sample size from the studies ranged from 357 to 4406 with a total of 32158 observations.

**Figure 3:**
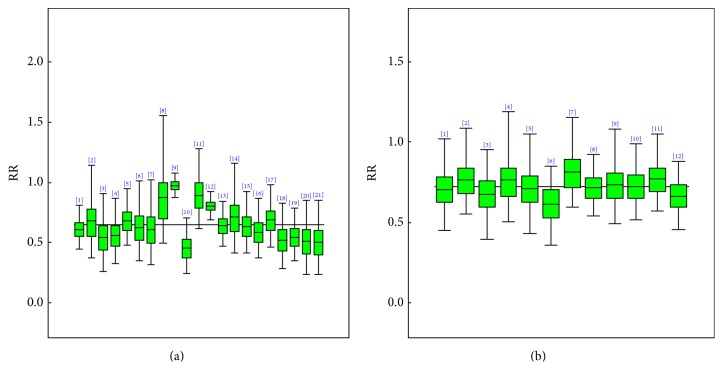
Posterior box plots describing the 95% credible intervals for each study's specific odds ratio (OR) estimate where (a) represents high and (b) represents moderate against no/low physical activity for Alzheimer's disease.

As presented in Table 2 and Figure 3, there is a negative association between high and moderate against no/low physical activity with Alzheimer's disease. The overall or grand mean effect (odds ratio) and credible interval for participants classified under the category of highest physical activity (PA) when compared with the no/lowest were 0.62 and 95% CI of (0.49, 0.75). The grand mean for moderate PA against no/lowest was 0.71 also with a 95% CI of (0.56, 0.89). Our analysis with the Copas selection model showed that the presence of publication bias was negligible. The estimated *ρ* values for both highest and moderate PA against no/lowest were 0.04 with a 95% CI of (−0.94, 0.97) and 0.04 with a 95% CI of (−0.93, 0.94), respectively. The posterior probabilities were also 0.53 and 0.54, respectively. There was negligible heterogeneity between studies, with either high or moderate against no/low PA. The estimated values for *τ* were 0.12, 95% CI (0.03, 0.32) for high, and 0.04, 95% (0.00, 0.21) for moderate. A very good number of sensitivity analyses were conducted and presented also in Table 2. It was observed from these analyses that men had a more protective effect of 39% as against women of 36%. In other words, the odds ratio for men was 0.61 with 95% CI (0.48, 0.75) and 0.64 with 95% CI of (0.49, 0.80) for women. A follow-up period of less than or equal to 5 years compared to those greater than 5 years had an approximately equal effect. For the sample size those with more than 1000 had a 40% reduced risk of Alzheimer's disease compared to those with less than 1000 36%.

### 3.3. Vascular Dementia

**Figure 4:**
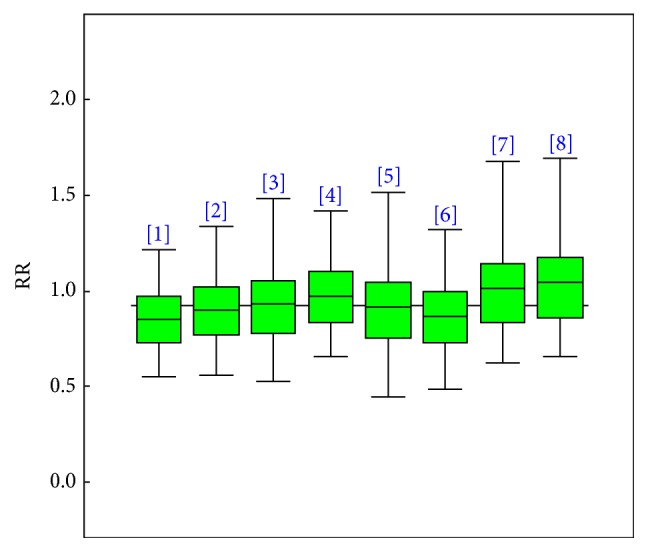
Posterior box plots describing the 95% credible intervals for each study specific odds ratio (OR) estimate for high against no/low physical activity for vascular dementia.

There were a total of 7 prospective and 8 cohort studies that assessed the effect of physical activity on vascular dementia. All the studies that examined vascular dementia also did the same for either all-cause dementia or Alzheimer's disease. The results obtained showed a nonsignificant association between physical activity and vascular dementia as also depicted in Figure 4. The posterior odds ratio and a 95% credible interval of 0.92 and (0.62, 1.30) were obtained. The Copas selection model showed a negligible presence of publication bias. The posterior estimate for *ρ* is −0.02 with a 95% CI of (−0.96, 0.96) with a posterior probability of 0.48 and that of *τ* is 0.07, 95% CI (0.00, 0.40).

### 3.4. Cognitive Decline

Cognitive decline was examined from baseline and on subsequent visits with Mini Mental State Examination (MMSE), Modified Mini Mental State Examination (3MS), and/or Clifton Assessment Procedures for the Elderly (CAPE). 18 prospective studies were retrieved with 22 cohorts for high or vigorous against low or no physical activity out of which 9 prospective studies with 11 cohorts examined and obtained results for moderate against low or no PA. The sample from the individual studies ranged from 37 to 10,308.

**Figure 5:**
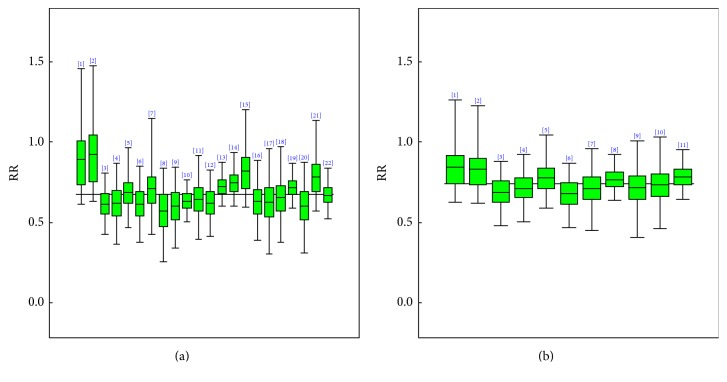
Posterior box plots describing the 95% credible intervals for each study's specific odds ratio (OR) estimate where (a) represents high and (b) represents moderate against no/low physical activity for cognitive decline.

Figure 5 and Table 3 contain the overall estimates for highest and moderate as against no/lowest physical activities as well as the sensitivity analysis that were carried out. A negative association for high and moderate physical activities compared with no/lowest was observed for cognitive decline. The grand mean effect and credible interval for highest physical activity (PA) compared with the no/lowest were 0.67 (0.55, 0.78). The overall mean effect and its credible interval for moderate against no/low PA were 0.74 (0.60, 0.90). The Copas selection model showed that there was no publication bias that may affect the posterior mean effect size estimate. The estimated *ρ* for both highest and moderate against no/lowest PA were 0.09 with a 95% CI of (−0.91, 0.94) and 0.05 with a 95% CI of (−0.93, 0.97), respectively. The posterior probabilities were also 0.57 and 0.54, respectively. Heterogeneity was not significantly present among studies; *τ* was estimated to be 0.06, 95% CI (0.00, 0.24) for high and 0.04, 95% CI (0.00, 0.20) for moderate. A similar number of sensitivity analyses as done for Alzheimer's disease and dementia were conducted and presented in Table 3. We observed a more protective effect of 35% for women and men of 29%, with odds ratio for women 0.65 (0.51, 0.76) 95% CI and 0.71 with 95% CI of (0.58, 0.86) for men. A follow-up period of less than or equal to 5 years compared to those greater than 5 years had odds ratio of 0.63, 95% CI (0.52, 0.74) and odds ratio of 0.84, 95% CI (0.38, 1.53), respectively. For a sample size less than or equal to 1000, a 41% reduced risk of cognitive decline was observed compared to 29% for a sample greater than 1000. Elderly individuals above the age of 65 are likely to reduce the risk of developing cognitive decline of 36% by staying highly physically active.

## 4. Discussion

In this research, two different models were applied to the meta-analysis of cognitive decline, dementia, and its subtypes. They are the Copas selection model (to account for publication bias) and the Dirichlet process model (to control for heterogeneity). The Copas model is based on conducting a number of sensitivity analyses by assigning different values to its parameters within the range of 0.01 to 0.99. These values are chosen purely on the subjective judgement of the researcher on the bases of perceived knowledge of publication bias. Most often, interpretations of estimates from these assumed values become somewhat confusing as a result of obtaining more than one posterior estimate. In other to overcome this issue with the Copas model via the Bayesian approach, we introduced what is referred to as* triangular prior*. The* triangular prior* as illustrated in the methods section is developed to account for the probability of publication for both* low* and* large* assuming information from experts; historical data or personal judgement is not available or appropriate.

Lower and upper bound for the probability of publication represented by *P*_low_ and *P*_large_ of studies having either large or small standard errors were specified following [51]. Since the Copas selection model parameters are not straightforwardly interpretable, it is rather more appropriate and easier specifying our prior knowledge on the probability of publication than on the parameters directly [51, 59]. The probability of publication is directly related to the study specific effect size and its corresponding standard errors, implying that studies with large standard errors have small probabilities of either being published or accepted for publication while those with small standard errors have large probabilities of being published or accepted for publication.

The overall number of observations for all-type dementia, Alzheimer's disease, vascular dementia, and cognitive decline included in this study was 117410 with 32, 21, 8, and 22 cohorts, respectively. The posterior estimates showed that both high and moderate physical activities are inversely related to dementia; that is, the risk of developing dementia is reduced by about 21% for high PA and 24% for moderate PA. Studies carried out by [36, 39, 60] support our findings. A similar meta-analysis conducted by Hamer and Chida [27] and Blondell et al. [28] revealed 28% higher and 16% lower benefit, respectively, in relation to high PA for dementia as compared to the findings in this current research. The findings from this study are more reliable and accurate than the previous findings. This is so because this meta-analysis involved more cohorts, 32 as compared to that of Hamer and Chida [27] with 14 cohorts, and did not also make any assumptions of normality. There are other studies suggesting that physical activity irrespective of whether it is high or moderate does not significantly reduce the risk of dementia; these include [22, 23, 35, 37].

Though physical activity reduces the risk of dementia, its benefit is higher for Alzheimer's disease among high and moderate, that is, 38% and 29%, respectively. From the sensitivity analysis, males are more likely to have 39% reduced risk of developing Alzheimer's disease compared to women of 36% unlike dementia where there is just a 1% difference between the sexes. With vascular dementia, there is no statistically significant risk reduction of high against low/no physical activity. These findings suggest that other modifiable risk factors may need to be considered. The limitation to the vascular dementia findings may be due to the number of cohorts (8) in this study. Subgroup analyses were not conducted for vascular dementia as was done for the others, the reason being the number of studies (for instance, 2 or a maximum of 3) for each group.

A meta-analysis conducted by Sofi et al. [26] suggests that high and moderate PA reduce the risk of cognitive decline by about 38% and 35%. The findings from our analysis with a high power (22 cohorts) support that of Sofi et al. [26] but disagree with the percentage reduction. High physical activity reduces the risk of cognitive decline by about 33% and that of moderate by about 26% in this current analysis. Sofi et al. [26] analysis did not account for the presence of heterogeneity as done in this current study via the adoption of a Bayesian nonparametric (Dirichlet process) model. The sensitivity analysis showed a much significant difference for estimates of association in relation to participants age groupings with a 34% for ≥65 and 21% for <65 years old. Further, we saw a 41% reduced risk for sample <1000 as compared to 39% for ≥1000.

There are a number of explanations given with respect to the protective effect of physical activity on a person's general health. Findings from Kalmijn et al. [61] suggest that people who have very active lifestyles are able to reduce their cortisol levels that help prevent stress. This also has a positive effect on cognitive function. Chodzko-Zajko and Moore [62] reveal that physical fitness helps to protect against cerebrovascular integrity, in that it sustains the flow of blood to the brain as well as supply oxygen and other nutrients to it; see also [63, 64].

Some of the limitations that might have had an effect on our study findings were the following. (1) The inclusion of studies was conducted over 30 years, at which period the studies differed substantially in terms of their sample size, sampling technique, methods, and their reporting abilities. (2) Combined results are reported in the literature with respect to sex. Some of the records retrieved did not differentiate or analyse their data according to sex (male and/or female) and therefore findings reported had an overall estimate representing both sexes; hence, the subgroup analysis for sex may not be representative. (3) Studies that were included in this review measured their primary or secondary variable using self-reported physical activity techniques and this may be prone to bias due to inaccurate reporting by interviewees either covertly or overtly which could have led to an underestimation or overestimation of the effect size. Though almost all the individual studies used self-reported questionnaires to obtain information on physical activity, the categorisation of this variable was not homogeneous across all the studies and this might have also introduced some level of bias in our findings. Therefore, interpretation of these findings must be made with caution.

## 5. Conclusion

The overall results suggest that physical activity is a beneficial or an important modifiable risk factor for reducing the risk of Alzheimer's disease, all-cause dementia, and cognitive decline but not vascular dementia. Physical activity is even more beneficial for Alzheimer's disease which accounts for about 60–70% of dementia cases. The results further reveal that moderate physical activity may be enough for reducing the risk of all-cause dementia. Our findings further reveal a more risk reduction of Alzheimer's disease for men who engage in physical activity compared to women but physical activity reduces the risk of cognitive decline for women than for men.

## Supplementary Material

The characteristics of interest upon which data were extracted are provided in the following tables according to authors and year of publications, countries of prospective studies, years of study, sample sizes, sexes or gender, follow-up years, ages of the participants at baseline, methods used to assess Alzheimer's disease, dementia, cognitive decline, and physical activity. The methodological quality of each of the studies that met the inclusion criterion were also appraised using Meta-Analyses of Statistics Assessment and Review Instrument (JBI-MAStARI). Using this instrument, studies that scored ≥7 were deemed to be of high quality, those within 4 ≥ studies ≤6 were judged to be of moderate quality, and that of scores <4 were of low quality.

## Figures and Tables

**Table 1:** Posterior summaries of the odds ratio, publication bias, heterogeneity, posterior probabilities, and estimated number of studies of physical activity on dementia with different sensitivity analysis.

Model	OR	*ρ*	PP_*ρ*_	*τ*	PS	ENS	*n*	f-up
HPA	0.79	0.07	0.55	0.05	32	52	46909	260.7
	(0.69, 0.88)	(−0.94, 0.96)		(0.01, 0.13)		(33.68, 63.8)		
MPA	0.76	0.13	0.59	0.06	15	20	20771	104.6
	(0.61, 0.94)	(−0.90, 0.97)		(0.00, 0.22)		(16.91, 28.35)		
HPAm	0.77	0.10	0.58	0.03	27	47	41225	255.6
	(0.68, 0.86)	(−0.93, 0.96)		(0.00, 0.09)		(28.73, 61.5)		
HPAf	0.78	0.02	0.51	0.07	20	24	35866	211.6
	(0.64, 0.91)	(−0.93, 0.95)		(0.01, 0.21)		(21.57, 30.23)		
HPA > 5	0.80	0.02	0.52	0.08	19	23	27384	216.7
	(0.66, 0.94)	(−0.95, 0.96)		(0.01, 0.22)		(20.33, 30.32)		
HPA ≤ 5	0.78	−0.05	0.46	0.06	13	17	19525	50
	(0.61, 0.92)	(−0.94, 0.91)		(0.00, 0.26)		(11.35, 23.33)		
HPA > 1000	0.79	0.04	0.52	0.06	17	21	37374	168.7
	(0.65, 0.93)	(−0.94, 0.96)		(0.01, 0.19)		(20.41, 27.45)		
HPA ≤ 1000	0.78	0.03	0.53	0.09	15	20	9535	108
	(0.61, 0.95)	(−0.94, 0.94)		(0.00, 0.30)		(17.02, 25.62)		
HPA ≥ 65	0.74	0.06	0.55	0.03	24	33	30980	157.7
	(0.63, 0.83)	(−0.92, 0.94)		(0.00, 0.13)		(25.49, 37.77)		
HPA < 65	0.94	−0.03	0.48	0.07	8	12	15929	103
	(0.72, 1.17)	(−0.94, 0.94)		(0.00, 0.04)		(9.31, 17.19)		

OR = odds ratio, *τ* = between-study variance, *ρ* = publication bias, PP_*ρ*_ = posterior probability of publication bias, PS = number of published studies, ENS = estimated number of studies, HPA = high physical activity, MPA = moderate physical activity, m = males, f = females, *n* = total sample size, and f-up = total follow-up time.

**Table 2:** Posterior summaries of the odds ratio, publication bias, heterogeneity, posterior probabilities, and estimated number of studies of physical activity on Alzheimer's disease with different sensitivity analysis.

Model	OR	*ρ*	PP_*ρ*_	*τ*	PS	ENS	*n*	f-up
HPA	0.62	0.04	0.53	0.12	21	25	32057	147
	(0.49, 0.75)	(−0.94, 0.97)		(0.03, 0.32)		(22.41, 31.89)		
MPA	0.71	0.04	0.54	0.04	12	17	15326	65.3
	(0.56, 0.89)	(−0.93, 0.94)		(0.00, 0.21)		(14.11, 23.14)		
HPAm	0.61	0.03	0.52	0.11	19	23	28738	147
	(0.48, 0.75)	(−0.94, 0.95)		(0.02, 0.31)		(20.42, 29.87)		
HPAf	0.64	−0.02	0.49	0.11	15	19	24847	135.9
	(0.49, 0.80)	(−0.95, 0.94)		(0.02, 0.36)		(16.43, 27.42)		
HPA > 5	0.62	0.03	0.48	0.30	11	15	15140	104.3
	(0.40, 0.88)	(−0.96, 0.95)		(0.05, 0.99)		(12.28, 25.88)		
HPA ≤ 5	0.63	−0.00	0.50	0.07	10	14	16917	42.7
	(0.46, 0.80)	(−0.95, 0.94)		(0.00, 0.35)		(11.35, 23.33)		
HPA > 1000	0.64	0.07	0.45	0.17	10	14	24254	70.7
	(0.42, 0.86)	(−0.96, 0.94)		(0.01, 0.65)		(13.32, 22.37)		
HPA ≤ 1000	0.60	0.06	0.55	0.19	11	16	7911	76.3
	(0.41, 0.83)	(−0.92, 0.95)		(0.00, 0.72)		(12.91, 21.99)		

OR = odds ratio, *τ* = between-study variance, *ρ* = publication bias, PP_*ρ*_ = posterior probability of publication bias, PS = number of published studies, ENS = estimated number of studies, HPA = high physical activity, MPA = moderate physical activity, m = males, f = females, *n* = total sample size, and f-up = total follow-up time.

**Table 3:** Posterior summaries of the odds ratio, publication bias, heterogeneity, posterior probabilities, and estimated number of studies of physical activity on cognitive decline with different sensitivity analysis.

Model	OR	*ρ*	PP_*ρ*_	*τ*	PS	ENS	*n*	f-up
HPA	0.67	0.09	0.57	0.06	22	28	38343	110.4
	(0.55, 0.78)	(−0.91, 0.94)		(0.00, 0.24)		(24.93, 33.18)		
MPA	0.74	0.05	0.54	0.04	11	16	27596	55.5
	(0.60, 0.90)	(−0.93, 0.96)		(0.00, 0.20)		(13.07, 22.29)		
HPAm	0.71	0.03	0.51	0.06	17	22	27927	90.4
	(0.58, 0.86)	(−0.91, 0.95)		(0.00, 0.24)		(19.61, 27.99)		
HPAf	0.65	0.08	0.56	0.04	15	21	33984	86.6
	(0.51, 0.76)	(−0.91, 0.95)		(0.00, 0.21)		(17.98, 27.0)		
HPA > 5	0.84	−0.03	0.48	0.56	6	10	8682	65
	(0.38, 1.53)	(−0.94, 0.94)		(0.02, 2.51)		(7.33, 17.99)		
HPA ≤ 5	0.63	0.10	0.58	0.02	16	21	29661	45.4
	(0.52, 0.74)	(−0.92, 0.94)		(0.00, 0.12)		(18.62, 27.48)		
HPA > 1000	0.71	0.04	0.53	0.09	11	16	34421	55
	(0.54, 0.89)	(−0.92, 0.95)		(0.00, 0.40)		(13.77, 22.76)		
HPA ≤ 1000	0.59	0.02	0.52	0.19	11	15	3922	55.4
	(0.38, 0.82)	(−0.93, 0.93)		(0.00, 0.84)		(12.68, 21.63)		
HPA ≥ 65	0.64	0.09	0.56	0.05	16	21	21342	81.4
	(0.50, 0.77)	(−0.90, 0.94)		(0.00, 0.02)		(18.26, 27.27)		
HPA < 65	0.79	−0.01	0.49	0.40	6	10	17001	29
	(0.43, 1.35)	(−0.95, 0.96)		(0.02, 1.86)		(7.87, 15.33)		

OR = odds ratio, *τ* = between-study variance, *ρ* = publication bias, PP_*ρ*_ = posterior probability of publication bias, PS = number of published studies, ENS = estimated number of studies, HPA = high physical activity, MPA = moderate physical activity, m = males, f = females, *n* = total sample size, and f-up = total follow-up time.
