# Supplementary material for: Impact of Physical Activity on Cognitive Decline, Dementia, and Its Subtypes: Meta-Analysis of Prospective Studies
Source: Biomed Res Int. 2017 Feb 7;2017:9016924. doi: 10.1155/2017/9016924 (PMC5320071; doi:10.1155/2017/9016924)
Supplement: Supplementary file 1 — The characteristics of interest upon which data were extracted are provided in the following tables according to authors and year of publications, countries of prospective studies, years of study, sample sizes, sexes or gender, follow-up years, ages of the participants at baseline, methods used to assess Alzheimer's disease, dementia, cognitive decline, and physical activity. The methodological quality of each of the studies that met the inclusion criterion were also appraised using Meta-Analyses of Statistics Assessment and Review Instrument (JBI-MAStARI). Using this instrument, studies that scored ≥7 were deemed to be of high quality, those within 4 ≥ studies ≤6 were judged to be of moderate quality, and that of scores <4 were of low quality. [file 9016924.f1.pdf]

Supplementary Materials

Table 4: Characteristics of the studies included in meta-analysis for physical activity on Alzheimer's disease and Dementia

(Author year)	Country	Sample	Gender	F-up-yrs	Age	Outcome Measure	Exposure Measure	phys. act.	Type
[22]	Sweden (1987-88)	732	M/F	6.4	$\geq$ 75	Clinical examination by a physician and psychological test according to DSM-III Edition, revised criteria	Questionnaire based on face-to-face interview on the levels of frequency of exercises; swimming gymnastics & walking	Not active<br>Less than daily<br>Daily	D
[23]	Japan (1988)	803	M/F	17	$\geq$ 65	The team comprising of stroke physicians & psychiatrist used 3 types of scales; HDS, HDSR & MMSE according to DSM-III	Self-reported Questionnaire based on the frequency on levels of exercise; cycling, jogging, dancing, hunting	Inactive<br>Active	D<br>AD<br>VaD
[31]	Canada (1991-92)	1831	M	5	$\geq$ 65	Screening was done based on clinical evaluation of study participants according DSM-IV criteria & NINCDS, ADRDA NINCDS-AIREN	Self-administered questionnaire on the intensity on levels of physical activities	None<br>Low<br>Medium<br>High	D<br>AD<br>VaD
[31]	Canada (1991-92)	2784	F	5	$\geq$ 65	Screening was done based on clinical evaluation of study participants according DSM-IV criteria & NINCDS, ADRDA NINCDS-AIREN	Self-administered questionnaire on the intensity on levels of physical activities	None<br>Low<br>Medium<br>High	D<br>AD
[63]	Japan (1985)	828	M/F	7	$\geq$ 65	The screening was done with DSM-III-R, NINCDS & ADRDA	Questionnaire based on face-to-face interview on the levels of frequency & intensity of PA	Inactive<br>Active	AD<br>VaD
[18]	Finland (1998)	1449	M/F	21	65-75	Diagnoses were made based on the DSM-III criteria and that of NINCDS & ADRDA for AD	Self-reported frequency or how regular they were involved in exercises per week	Inactive<br>Active	D<br>AD
[32]	Australia (1988-89)	1233	M	16	$\geq$ 60	Screening was done by geriatric team using computer tomography scanner	Admin. questionnaire about exercise frequency; walking, & sporting activities	Rarely $<$ weekly<br>Daily or weekly	D
[32]	Australia (1988-89)	1572	F	16	$\geq$ 60	Screening was done by geriatric team using computer tomography scanner	Admin. questionnaire about exercise frequency; walking, & sporting activities	Rarely $<$ weekly<br>Daily or weekly	D
[33]	UK (1984-88)	1005	M	16	56	Diagnoses were made based on the DSM-IV & NINDS-AIREN criteria with NINCDS & ADRDA	Self-reported frequency of PA for both work leisure activities	Low<br>Moderate<br>High	D

[64]	USA (1999)	357	M/F	5.2	≥ 65	Diagnoses was based on DSM-III-R with NINCDS & ADRDA	Information about frequency and intensity of exercise were collected from participants	No<br>Some<br>High	AD
[65]	USA (2005)	716	M/F	4	81.6	Participants were diagnosed by a clinician with NINCDS & ADRDA	Self-reported assessment of exercises adapted from the 1985 NHIS based on walking, running swimming on hrs/per week	No<br>Yes	AD
[34]	USA (1980-83)	469	F	5.1	≥ 75	A selected number of experts; neurologist, neuropsychologist, geriatric nurse clinician assessed participants with the using DSM-III-R	Information about the the frequency of exercise at baseline concerning; golfing, playing tennis, bicycling, walking were collected	< 9 points<br>$9 - 16$ points<br>> 16 points	D
[19]	USA (1991-93)	2257	M	5	71-93	Screening of participants was based on CASI and DSM-III-R, criteria	Self-administered questionnaire that obtained information about the average amount of distance walked per day	< 0.25 miles/day<br>$0.25 - 1$ miles/day<br>>1-2 miles/day<br>> 2 miles/day	D<br>AD<br>VaD
[66]	USA (1989-90)	3375	M/F	5	> 65	Screening of participants was based on 3MS, neuropsychiatric evaluation regardless of dementia risk & NINCDS-ADRDA	Trained interviewers at baseline used MMLTAQ and obtained information from participants about frequency of of exercise	$0 - 1$ activity<br>2 activities<br>3 activities<br>≥ 4 activities	D<br>AD<br>VaD
[20]	USA (1994-96)	1740	M/F	6.2	> 65	Screening of participants was based on CASI	Self-administered questionnaire about the frequency of exercises performed a day per/week for at least 15 minutes	< 3 times/week<br>≥ 3 times/week	D<br>AD
[35]	USA (1991-92)	580	M	6.1	> 65	Screening of participants was based on CASI, for both first and second evaluation and also with IWCODE with low cognition	Average number of hours spent in different levels of activities for 24 hours exercising	Low<br>Moderate<br>High	D<br>AD
[35]	USA (1991-92)	712	M	6.1	> 65	Screening of participants was based on CASI, for both first and second evaluation and also with IWCODE with moderate cognition	Average number of hours spent in different levels of activities for 24 hours exercising	Low<br>Moderate<br>High	D<br>AD
[35]	USA (1991-92)	971	M	6.1	> 65	Screening of participants was based on CASI, for both first and second evaluation and also with IWCODE with high cognition	Average number of hours spent in different levels of activities for 24 hours exercising	Low<br>Moderate<br>High	D<br>AD

[67]	USA (1992)	808	M/F	5	> 71	Screening of participants for incident dementia was by panel of experts; neurologist, neuropsychologist, geropsychiatrist	Information about vigorous exercise per week based on bicycling aerobics, running, sports & heavey work	No<br>Yes	D
[25]	Iceland (1965)	4761	M/F	26	51	Participants were examined by neurologist neupsychologist, on neuroradiologist according to DSM-IV-R	Regular exercise was asked participants and further questions about how number of times/week they exercised	None<br>$\leq$ 5 hours<br>$>$ 5 hours	D
[24]	Nerthaland (1990)	4406	M/F	14	> 61	Screened of dementia at baseline and follow-up using threes step protocol like MMSE, GMS ogrganic level & CAMDEX	The Zutphen Physical activity questionnaire was used to assess participants levels of of exercises in hours per week	No<br>Yes	D<br>AD
[68]	Nigeria (2003-4)	1408	M/F	3	> 65	10-Word Delayed Recall Test and a clinician home-based interview was implored with the help of DSM-IV	Questionnaire was use to collect information across the domain of transportation leisure-time and work	Low<br>Moderate<br>High	D
[21]	South Korea (2001)	578	M/F	2.4	≥ 65	Assessment was carried out using IADL, the CDRS, collateral information on past history, physical & neurological evaluation	A self-rated PA carried out at baseline on a 4-point scale	Not at all active<br>Not very active<br>Fairly active<br>very active	D
[69]	USA (1992)	1880	M/F	5.4	> 65	Screening for dementia was made according to DSM-III-R criteria by neuropsychologist and neurologist	A questionnaire was administered to and information about their frequency of exercises obtained and categorised	No<br>Some<br>Much	AD
[70]	Australia (1988)	2805	M/F	16	> 60	Information of the were collected by clinicians in hospitals and with follow-up questionnaires	Self-administered questionnaire on the frequency of exercises performed daily	Rarely<br>Daily	D
[71]	Italy (1999-00)	749	M/F	4	> 65	A standardized Screening was implored and an extensive neuropsychological assessment carried out others included GDS, MDB, DSM-IV-R NINCDS, ADRDA	Self-administered The Paffenbarger questionnaire was used to measure PA	Low<br>Moderate<br>Vigorous	D<br>AD<br>VaD
[72]	USA (2003)	587	M/F	3	≥ 90	Screened of dementia at baseline and follow-up using trained physicians & nurse practitioners & a neurologist & CASI-short & DSM-IV	Information of PA was based on the frequency of exercises per day on swimming, biking, fishing, jogging	Rarely<br>Frequently	D

[36]	USA (1989)	1772	M	7	> 65	Screening of participants was based on BMICT, BDRS & SEADLS. Diagnosis was carried out by physicians neuropsychologist	Information about participants involvement in PA was based on sporting events walking & visiting friends & relatives	Low<br>High	D
[73]	USA (1994)	801	M/F	5	> 71	Certified neurologist screened participants using NINCDS & ADRDA criteria & changes of specific measures of cognition	Information about participants frequency & intensity per day, week month & year were obtained	Quartile 1<br>Quartile 2<br>Quartile 3<br>Quartile 4	AD
[37]	USA (1967)	147	M	15	44.7	Participants were screened based on IQCODE & with Dementia questionnaire by a research nurse & psychometrician	Regular & frequency of exercises by by participants were collected using questionnaires	Low<br>High	D
[30]	Finland (1972)	859	F	28	78.8	Participants went through detailed neurological, neuropsychological examinations and DSM-III, NINCDS & ADRDA criteria	Assessment was made based on frequency & intensity exercises lasting about 20-30 mins	Low<br>Moderate<br>High	D<br>AD
[30]	Finland (1972)	535	M	28	78.8	Participants went through detailed neurological, neuropsychological examinations and DSM-III, NINCDS & ADRDA criteria	Assessment was made based on frequency & intensity exercises lasting about 20-30 mins	Low<br>Moderate<br>High	D<br>AD
[74]	Germany (2003)	2492	M/F	4.5	> 75	Agreed at consensus conferences the included by geriatrician or geriatric psychiatrist using DSM-IV, NINCDS & ADRDA criteria	Physical activities were based on biking, cycling, golfing, swimming, hiking	No<br>Yes	D<br>AD
[75]	France (1988)	2040	M/F	3	> 65	A standardized Screening was implored with the Hachinski score & DSM-III-R, NINCDS, ADRDA criteria	Self-administered questionnaire was used to collect PA information	No<br>Yes	D<br>AD
[57]	Finland (1972)	1449	M/F	21	65-79	A standardized Screening was implored with the DSM-III-R, NINCDS, & ADRDA criteria	Self-administered questionnaire based on the frequency & intensity of PA from community & occupation	Sedentary<br>Active	D

NINCDS-ADRDA: National Institute of Neurological and Communicative Disorders and Stroke and the Alzheimers Disease and Related Disorders Association; NINDS-AIREN: National Institute of Neurological and Communicative Disorders and Association Internationale pour la Recherche et l'Enseignement en Neurosciences; NSAIDs: Non-Steroidal Anti-Inflammatory Drugs; PA: Physical Activity; VaD: Vascular Dementia; VCI: Vascular Cognitive Impairment; : Dementia; DSM: Diagnostic and Statistical Manual of Mental Disorders; AD: Alzheimers Disease; CAPE: Clifton Assessment Procedure for the Elderly; CDR: Clinical Dementia Rating scale

Table 5: Characteristics of the Studies included in meta-analysis for physical activity & cognitive decline

Source	Country	Sample	Gender	F-up-yrs	Age	Outcome Measure	Exposure Measure	Cat. of phys. act.	Type
[33]	UK (1984-88)	1005	M	16	56	Diagnoses were made based on clinical assessment & CAMCOG	Self-reported frequency of PA for both work leisure activities	Low<br>Moderate<br>High	CIND
[76]	Germany (2001-3)	3903	M/F	2	>55	Screening for cognitive impairment was based 6-CIT	Screening was obtained based on participants frequency performing PA	No<br>Moderate<br>High	CI
[77]	USA (1987)	1146	M/F	4	$\geq$ 65	MMSE	Information about frequency and duration where $\geq$ 3 times per week for $\geq$ 30 mins considered high, $<$ 30 low and no-exercise	None<br>Low<br>High	CD
[31]	Canada (1991)	1831	M	5	$\geq$ 65	3MS score based on $\leq$ 77 and $\geq$ 78. Taking through an standard 3-stage clinical evaluation and final decisions made via concensus conference b/n physicians & newropsychometrist	Self-administered questionnaire on the intensity of their levels of physical activities	None<br>Low<br>Medium<br>High	CIND
[31]	Canada (1991)	2784	F	5	$\geq$ 65	3MS score based on $\leq$ 77 and $\geq$ 78. Taking through an standard 3-stage clinical evaluation and final decisions made via concensus conference b/n physicians & newropsychometrist	Self-administered questionnaire on the intensity of their levels of physical activities	None<br>Low<br>Medium<br>High	CIND
[38]	USA (1989)	39	M	4.7	$\geq$ 85	MMSE & CDR	Self-reported using personal and family history questionnaire based walking, biking, dancing golfing, running & jogging	$>$ 4 hours a week	CI
[38]	USA (1989)	37	F	4.7	$\geq$ 85	MMSE & CDR	Self-reported using personal and family history questionnaire based walking, biking, dancing golfing, running & jogging	$\leq$ 4 hours a week<br>$>$ 4 hours a week	CI
[78]	USA (2000)	150	M/F	8	$\geq$ 60	MMSE	Information about time spent in exercising for the past 12 months Japanese lifestyle monitoring questionnaire	None<br>Level 2<br>Level 3	CD
[79]	UK (1985-88)	10308	M/F	1	46-68	Screening for cognition was by cognitive battery test of 5 standard task	Screening was obtained based on participants frequency & duration in PA	No<br>Moderate<br>High	CI
[39]	China (1999)	469	F	3	$\geq$ 70	Information/Orientation part of the Clifton assessment for the elderly using a cut-off point of 7	Questionnaire based on face-to-face interview on the levels of exercise	No<br>Yes	CI

[39]	China (1999)	519	M	3	≥ 70	Information/Orientation part of the Clifton assessment for the elderly using a cut-off point of 7	Questionnaire face-to-face interview on levels of exercise	No<br>Yes	CI
[40]	USA (1986)	5925	F	8	≥ 65	MMSE	Self-reported blocks walked per week and by total kilocalories expended per week in recreation	1st quartile<br>2nd quartile<br>3rd quartile<br>4th quartile	CD
[80]	Singapore (2004-5)	1635	M/F	1-2	$\geq$55	Diagnoses were made using MMSE	Self-reported frequency of PA in different categories	No<br>Yes	CD
[81]	USA (1980-83)	401	M/F	21	$75 - 85$	They administered three test to all participants; digit symbol substitution, digit span (total span) & category of fluency test	Self-reported information on frequency and intensity of exercise	Lowest<br>Highest	VCI
[41]	Australia (1996)	618	M	4.8	≥ 65	MMSE	Self-reported questionnaire on the frequency & intensity about levels of exercise	Non-vigorous<br>Vigorous	CI
[82]	USA (1998-99)	197	M/F	3.5	$\bar{74}.8$	Middleton modified mini-mental state examination	The modified version of Minnesota leisure time act questionnaire on duration & intensity	No<br>Yes	CI
[42]	Nertherlands (1990)	347	M	3	≥ 65	MMSE	Self-administered questionnaire on frequency & duration of walking, bicycling hobbies and gardening for previous week obtained	$\leq$30 mins a day<br>31 - 60 mins a day<br>>60 mins a day	CD
[83]	USA (1991)	4683	M/F	5	≥ 65	3MS & clinical Evaluation	Self-administered questionnaire that obtained information on individual level intensity and frequency of their physical act.	Low<br>Moderate<br>High	CIND
[43]	Italy (1999)	1201	F	12	70 - 75	MSQ	Administered questionnaire about their walking distance with $<$ 2km considered low and $\geq$ 2km high	Low<br>High	CD
[84]	Japan (2002)	567	M/F	5	$70 - 84$	MMSE	Information about jogging, walking, dancing japanese croquet, running & gymnastics were collected	No<br>Yes	CD
[21]	South Korea (2001)	578	M/F	2.4	≥ 65	Assessment was carried out using IADL & MMSE	A self-rated PA carried out at baseline on a 4-point scale	Not at all active<br>Not very active<br>Fairly active<br>very active	D

MMSE: Mini Mental State Examination; 3MS:Modified-Mini Mental State Examination; MSQ: Mental Status Questionnaire; PA: Physical Activity; VaD: Vascular Dementia; VCI: Vascular Cognitive Impairment; : Dementia; CAPE: Clifton Assessment Procedure for the Elderly; CD: Cognitive Decline; CDR: Clinical Dementia Rating scale; CI: Cognitive Impairment; CIND: Cognitive Impairment No Dementia, IADL: instrumental activities of daily living scale

Table 6: PRISMA 2009 Checklist

**Section/topic**	**#**	**Checklist item**	**Reported on page #**
**TITLE**			
Title	1	Identify the report as a systematic review, meta-analysis, or both.	1
**ABSTRACT**			
Structured summary	2	Provide a structured summary including, as applicable: background; objectives; data sources; study eligibility criteria, participants, and interventions; study appraisal and synthesis methods; results; limitations; conclusions and implications of key findings; systematic review registration number.	1
**INTRODUCTION**			
Rationale	3	Describe the rationale for the review in the context of what is already known.	4
Objectives	4	Provide an explicit statement of questions being addressed with reference to participants, interventions, comparisons, outcomes, and study design (PICOS).	NA
**METHODS**			
Protocol and registration	5	Indicate if a review protocol exists, if and where it can be accessed (e.g., Web address), and, if available, provide registration information including registration number.	NA
Eligibility criteria	6	Specify study characteristics (e.g., PICOS, length of follow-up) and report characteristics (e.g., years considered, language, publication status) used as criteria for eligibility, giving rationale.	4-6
Information sources	7	Describe all information sources (e.g., databases with dates of coverage, contact with study authors to identify additional studies) in the search and date last searched.	Fig 1 & Suppl material
Search	8	Present full electronic search strategy for at least one database, including any limits used, such that it could be repeated.	6
Study selection	9	State the process for selecting studies (i.e., screening, eligibility, included in systematic review, and, if applicable, included in the meta-analysis).	4, Fig 1
Data collection process	10	Describe method of data extraction from reports (e.g., piloted forms, independently, in duplicate) and any processes for obtaining and confirming data from investigators.	6
Data items	11	List and define all variables for which data were sought (e.g., PICOS, funding sources) and any assumptions and simplifications made.	6
Risk of bias in individual studies	12	Describe methods used for assessing risk of bias of individual studies (including specification of whether this was done at the study or outcome level), and how this information is to be used in any data synthesis.	NA
Summary measures	13	State the principal summary measures (e.g., risk ratio, difference in means).	5
Synthesis of results	14	Describe the methods of handling data and combining results of studies, if done, including measures of consistency (e.g., I^2) for each meta-analysis.	9-12

Section/topic	#	Checklist item	Reported on page #
Risk of bias across studies	15	Specify any assessment of risk of bias that may affect the cumulative evidence (e.g., publication bias, selective reporting within studies).	9-12
Additional analyses	16	Describe methods of additional analyses (e.g., sensitivity or subgroup analyses, meta-regression), if done, indicating which were pre-specified.	6-8
**RESULTS**			
Study selection	17	Give numbers of studies screened, assessed for eligibility, and included in the review, with reasons for exclusions at each stage, ideally with a flow diagram.	Fig 1
Study characteristics	18	For each study, present characteristics for which data were extracted (e.g., study size, PICOS, follow-up period) and provide the citations.	Suppl Material
Risk of bias within studies	19	Present data on risk of bias of each study and, if available, any outcome level assessment (see item 12).	NA
Results of individual studies	20	For all outcomes considered (benefits or harms), present, for each study: (a) simple summary data for each intervention group (b) effect estimates and confidence intervals, ideally with a forest plot.	Fig 2-5
Synthesis of results	21	Present results of each meta-analysis done, including confidence intervals and measures of consistency.	Fig 2-5
Risk of bias across studies	22	Present results of any assessment of risk of bias across studies (see Item 15).	12-21
Additional analysis	23	Give results of additional analyses, if done (e.g., sensitivity or subgroup analyses, meta-regression [see Item 16]).	12-21
**DISCUSSION**			
Summary of evidence	24	Summarize the main findings including the strength of evidence for each main outcome; consider their relevance to key groups (e.g., healthcare providers, users, and policy makers).	24
Limitations	25	Discuss limitations at study and outcome level (e.g., risk of bias), and at review-level (e.g., incomplete retrieval of identified research, reporting bias).	24
Conclusions	26	Provide a general interpretation of the results in the context of other evidence, and implications for future research.	22-24
**FUNDING**			
Funding	27	Describe sources of funding for the systematic review and other support (e.g., supply of data); role of funders for the systematic review.	25

From: Moher D, Liberati A, Tetzlaff J, Altman DG, The PRISMA Group (2009). Preferred Reporting Items for Systematic Reviews and Meta-Analyses: The PRISMA Statement. PLoS Med 6: e1000097. doi:10.1371/journal.pmed1000097
For more information, visit: **www.prisma-statement.org**.

Table 7: Quality Assessment of Systematic Reviews and Meta-Analyses

**Criteria**	**Yes**	**No**	**Other(CD, NR, NA)***
1. Is the review based on a focused question that is adequately formulated and described?	✓		
2. Were eligibility criteria for included and excluded studies predefined and specified?	✓		
3. Did the literature search strategy use a comprehensive, systematic approach?	✓		
4. Were titles, abstracts, and full-text articles dually and independently reviewed for inclusion and exclusion to minimize bias?	✓		
5. Was the quality of each included study rated independently by two or more reviewers using a standard method to appraise its internal validity?	✓		
6. Were the included studies listed along with important characteristics and results of each study?	✓		
7. Was publication bias assessed?	✓		
8. Was heterogeneity assessed? (This question applies only to meta-analyses.)	✓		

Source: NIH- Systematic Evidence Reviews and Clinical Practice Guidelines Systematic Evidence Reviews Study Quality Assessment Tools Quality Assessment of Systematic Reviews and Meta-Analyses

Table 8: Methodological quality assessment

Critical appraisal for included studies of cognitive decline, Alzheimer's disease and Dementia

**First author, year of publication and reference number**	**Q1**	**Q2**	**Q3**	**Q4**	**Q5**	**Q6**	**Q7**	**Q8**	**Q9**	**Q10**	**Overall quality of the study**
Wang et al., 2002	N	Y	Y	Y	Y	N	Y	Y	Y	Y	8/10 (high)
Kishimoto, 2016	N	Y	Y	Y	Y	Y	Y	Y	Y	Y	9/10(high)
Laurin et al., 2001	Y	Y	Y	Y	Y	N	Y	Y	Y	Y	9/10 (high)
Yoshitake et al., 1995	N	Y	Y	Y	Y	N	Y	Y	Y	Y	8/10(high)
Rovio et al., 2005	Y	Y	Y	Y	Y	N	Y	Y	Y	Y	9/10(high)
Simons et al., 2006	N	U	Y	N	Y	N	Y	Y	Y	Y	6/10(moderate)
Morgan et al., 2012	N	Y	Y	Y	Y	N	Y	Y	Y	Y	8/10(high)
Scarmeas et al., 2011	Y	Y	Y	Y	Y	N	Y	Y	Y	Y	9/10(high)
Buchman et al., 2012	N	Y	Y	Y	Y	N	Y	Y	Y	Y	8/10(high)
Verghese et al., 2003	N	U	U	Y	Y	N	Y	Y	Y	Y	6/10(moderate)
Abbott et al., 2004	U	Y	Y	Y	Y	Y	Y	Y	Y	Y	9/10(high)
Podewils et al., 2005	Y	Y	Y	Y	Y	U	Y	Y	Y	Y	9/10(high)
Larson et al., 2006	Y	Y	Y	Y	Y	N	Y	Y	Y	Y	9/10(high)
Taafe et al., 2008	N	Y	Y	Y	Y	N	Y	Y	Y	Y	8/10(high)
Bowen, 2012	Y	Y	Y	Y	Y	N	Y	Y	Y	Y	9/10(high)
Chang et al., 2010	N	U	Y	Y	Y	U	Y	Y	Y	Y	7/10(high)
de Bruijn et al., 2013	N	Y	Y	Y	Y	Y	Y	Y	Y	Y	9/10(high)
Gureje et al., 2011	N	Y	Y	Y	Y	N	Y	Y	Y	Y	8/10 (high)
Kim et al., 2011	U	Y	Y	Y	Y	U	Y	Y	Y	Y	8/10(high)
Scarmeas et al., 2009	N	Y	Y	Y	Y	N	Y	Y	Y	Y	8/10(moderate)
McCALLUM, 2007	N	Y	Y	Y	Y	N	Y	Y	Y	Y	8/10(high)
Ravaglia et al., 2008	N	Y	Y	Y	Y	N	Y	Y	Y	Y	8/10(high)
Paganini-Hill, 2015	N	Y	Y	Y	Y	U	Y	Y	Y	Y	8/10(high)
Scarmeas et al., 2001	N	Y	Y	Y	Y	N	Y	Y	Y	Y	8/10(high)
Wilson et al., 2002	N	Y	Y	Y	Y	N	Y	Y	Y	Y	810(high)

Carlson et al., 2008	N	Y	Y	Y	Y	N	Y	Y	Y	Y	8/10(moderate)
Tolppanen, 2015	Y	Y	Y	Y	Y	N	Y	Y	Y	Y	9/10(high)
Luck et al., 2014	Y	Y	Y	Y	Y	N	Y	Y	Y	Y	9/10(high)
Fabrigoule, 1995	N	Y	Y	Y	Y	N	Y	Y	Y	Y	8/10(high)
Rovio et al., 2007	N	Y	Y	Y	Y	N	Y	Y	Y	Y	8/10(moderate)
Etgen et al., 2010	N	U	Y	Y	Y	U	Y	Y	Y	**Y**	7/10(high)
Lytle et al., 2004	Y	U	Y	Y	Y	N	Y	Y	Y	Y	8/10(high)
Sumic et al., 2007	N	N	Y	Y	Y	N	Y	Y	Y	Y	7/10(high)
Lee et al., 2013	N	N	Y	Y	Y	U	Y	Y	Y	U	6/10(moderate)
Singh-M et al., 2005	N	N	Y	Y	Y	N	Y	Y	Y	Y	7/10(high)
Ho et al., 2001	Y	U	Y	Y	Y	N	Y	Y	Y	Y	8/10(high)
Yaffe et al., 2001	N	Y	Y	Y	Y	Y	Y	Y	Y	Y	9/10(high)
Niti et al., 2008	N	U	Y	Y	Y	N	Y	Y	Y	Y	7/10(high)
Verghese et al., 2009	N	Y	Y	Y	Y	Y	Y	Y	Y	Y	9/10(high)
Flicker et al., 2005	N	U	Y	Y	Y	U	Y	Y	Y	Y	7/10(high)
Middleton et al., 2011	N	N	Y	Y	Y	N	Y	Y	Y	Y	7/10(high)
Schuit et al., 2001	N	N	Y	Y	Y	N	Y	Y	Y	Y	7/10(high)
Middleton et al., 2008	Y	Y	Y	Y	Y	Y	Y	Y	Y	Y	10/10(high)
Pignatti et al., 2002	U	N	Y	Y	Y	N	Y	U	Y	N	5/10(moderate)
Iwasa et al., 2012	N	Y	Y	Y	Y	N	Y	Y	Y	Y	8/10(high)

Criteria was adapted from the JBI Critical Appraisal Checklist for descriptive/case series research. **For the included studies**: 1) Was the study based on a random or pseudo-random sample? 2) Were the criteria for inclusion in the sample clearly defined? 3) Were confounding factors identified and strategies to deal with them stated? 4) Were outcomes assessed using objective criteria? 5) If comparisons were being made, was there sufficient description of the groups? 6) Were the outcomes of people who withdrew described and included in the analysis? 7) Were outcomes measured in a reliable way? 8) Was appropriate statistical analysis used? 9) Was the research ethical according to current criteria or, for recent evidence of ethical approval by an appropriate body? 10) Did the conclusions drawn in the research report flow from the analysis or interpretation of the data? Each item was rated Y = Yes, N = No or U = Unclear. Unclear was awarded where not enough information was provided. High quality: meets ≥ 7 criteria, Moderate quality: meets ≥ 4 criteria, Low quality: < 4 criteria.
